# Two genomic regions encoding exopolysaccharide production systems have complementary functions in *B. cereus* multicellularity and host interaction

**DOI:** 10.1038/s41598-020-57970-3

**Published:** 2020-01-22

**Authors:** Joaquin Caro-Astorga, Ana Álvarez-Mena, Jesús Hierrezuelo, Juan Antonio Guadix, Zahira Heredia-Ponce, Yohanna Arboleda-Estudillo, Elena González-Munoz, Antonio de Vicente, Diego Romero

**Affiliations:** 1 Instituto de Hortofruticultura Subtropical y Mediterránea “La Mayora” –Departamento de Microbiología, Universidad de Málaga, Bulevar Louis Pasteur 31 (Campus Universitario de Teatinos), 29071 Málaga, Spain; 2 Departamento de Biología Animal, Facultad de Ciencias, Universidad de Málaga - IBIMA, Campus de Teatinos s/n, 29071 Málaga, Spain; 3 Centro Andaluz de Nanomedicina y Biotecnología (BIONAND), Junta de Andalucía, Universidad de Málaga, C/ Severo Ochoa 35, 29590 Campanillas (Málaga), Spain; 4 LARCEL, Andalusian Laboratory of Cell Reprogramming, Andalusian Center for Nanomedicine and Biotechnology-BIONAND, 29590 Málaga, Spain

**Keywords:** Biofilms, Microbial ecology

## Abstract

Bacterial physiology and adaptation are influenced by the exopolysaccharides (EPS) they produce. These polymers are indispensable for the assembly of the biofilm extracellular matrix in multiple bacterial species. In a previous study, we described the profound gene expression changes leading to biofilm assembly in *B. cereus* ATCC14579 (CECT148). We found that a genomic region putatively dedicated to the synthesis of a capsular polysaccharide (*eps2*) was overexpressed in a biofilm cell population compared to in a planktonic population, while we detected no change in the transcript abundance from another genomic region (*eps1*) also likely to be involved in polysaccharide production. Preliminary biofilm assays suggested a mild role for the products of the *eps2* region in biofilm formation and no function for the products of the *eps1* region. The aim of this work was to better define the roles of these two regions in *B. cereus* multicellularity. We demonstrate that the *eps2* region is indeed involved in bacterial adhesion to surfaces, cell-to-cell interaction, cellular aggregation and biofilm formation, while the *eps1* region appears to be involved in a kind of social bacterial motility. Consistent with these results, we further demonstrate using bacterial-host cell interaction experiments that the *eps2* region is more relevant to the adhesion to human epithelial cells and the zebrafish intestine, suggesting that this region encodes a bacterial factor that may potentiate gut colonization and enhance pathogenicity against humans.

## Introduction

The biofilm extracellular matrix mainly consists of proteins, extracellular DNA and exopolysaccharides (EPSs). The relative importance of each component for biofilm formation depends on the species, but these three elements generally confer the diverse physico-chemical features defining the extracellular matrix^1^. EPSs are large molecules with complex structures and diverse chemical properties that cover a variety of functions relevant for bacterial physiology and multicellular lifestyles. Some of their functions include maintaining biofilm architecture, establishing colony morphology, and providing robustness to biofilms^2–5^. EPSs are also involved in the adhesion of cells to biotic and abiotic surfaces which, for instance, determines the ability of the bacterial cells to efficiently colonize natural niches, such as the plant rhizosphere^6–10^. Interactions among bacterial cells have also been shown to be supported by EPSs, which can induce cellular aggregation^11,12^. Thus, it is not surprising to find that EPSs are involved in the migration and mobility of bacterial colonies, functioning either as rails over which the bacteria move or in reducing friction and facilitating the displacement of either single cells or the entire community in the modes of social movement known as swarming and sliding, depending on what other bacterial factors are involved^13–17^. Roles for EPSs in stress resistance have also been widely reported, including conferring resistance to osmotic, desiccation, and oxidative stress and in cryoprotection^18–22^. In some cases, EPSs offer protection against phage attack, even though specific EPSs are sometimes the targets recognized and enzymatically degraded by phage capsid proteins to trigger infection^23–25^. In addition to their roles in conferring resistance to sanitizers, detergents, and antimicrobials and their implications in human disease, EPSs can also contribute to the corrosion of stainless steel, a material that is very commonly used in the food industry, tanks, pipes and ship hulls, on which *B. cereus* is prone to establish biofilms^26^.

The variety of EPS functions reflect differences in chemical composition, particularly in terms of the different sugar residues present, chemical bond types, ramifications, sugar modifications and chain length, all of which are determined via complex biosynthetic processes^27^. Therefore, common polysaccharides, like cellulose, can be produced by bacterial species in different genera, while others are strain specific^28,29^. The study of the bacterial factors involved in biofilm formation is useful for understanding the functions of the biofilm in bacterial physiology and ecology, including interactions between bacterial cells or their hosts. Furthermore, such studies can reveal potential bacterial targets for the development of strategies for minimizing the negative impacts of bacterial biofilms. Studies on *B. cereus* have highlighted the relevant contributions of EPSs to the total composition of the extracellular matrix, although their origins still remain uncertain^30^. Some reports have focused on the characterization of spore polysaccharides^31^ or secondary cell wall polysaccharide, both of which seem to be strain dependent^32^. In the phylogenetically-related species *B. subtilis*, the *epsA-O* operon encodes a group of enzymes required for the synthesis of biofilm EPS, and deletion of this operon leads to impaired biofilm formation^33,34^. *B. cereus* ATCC14579 possesses a homologous region (*Bc5279-Bc5263*, henceforth *eps1*), which we have confirmed to be irrelevant for biofilm formation in this strain, a finding that is also supported by studies of the strain *B. cereus* 905 in a pellicle model of biofilm^35^. However, it appears that deletion of some of the genes in this region impairs biofilm formation in the strain ATCC10987, using PEG plates as a model of biofilm^28^, a discordancy that reveals the heterogeneity that can exist among bacterial strains of the same species.

Prompted by an interest in the influences of EPS on bacterial physiology and ecology and the divergence in terms of their exact chemical composition and function, in this work we studied the structures of two genomic regions that putatively encode proteins that synthesize two different polysaccharides. As biofilm model, we used the ring of biomass adhered to the wall of the well of culture plates given that *B. cereus* 14579 only produce pellicles in specific conditions and long periods of incubation. We then explored the implications of these regions in *B. cereus* multicellularity and host interactions. We previously reported a transcriptomic analysis in which we showed that an additional region of the *B. cereus* ATCC14579 genome (containing the genes *Bc1583-Bc1591* and referred to as *eps2*) was overexpressed in biofilm cells compared to its expression level in planktonic cells^36^. This region has been annotated to be involved in capsular polysaccharide production, although *B. cereus ATCC14579* lacks a capsule. Our analysis indicates that the two putative polysaccharides play complementary roles in *B. cereus* multicellularity: while *eps1* promotes bacterial social mobility, *eps2* is involved in biofilm maturation, cell-to-cell interaction and aggregation. Interestingly, and consistent with these findings, EPS2 seems to be more important for the adhesion of *B. cereus* cells to human epithelial cells and to the zebrafish gut, a model used to study bacteria-gut interactions.

## Results

### The *eps1* and *eps2* regions are differentially expressed in *B. cereus* biofilm

A previous transcriptomic analysis of *B. cereus* cells grown under static conditions demonstrated that there were no statistically significant differences in the expression levels of a region homologous to the *B. subtilis eps*A-*O* operon (which is dedicated to EPS production) between biofilm and planktonic cells 24 and 48 h post-inoculation (Fig. Suppl. 1A). However, we found that a group of genes (*BC1583-BC1591*) absent in the *B. subtilis* group and annotated as “capsular polysaccharide biosynthesis” was upregulated in biofilm cells. Consistent with these findings, a *B. cereus eps1* mutant strain was not defective in biofilm formation, as demonstrated by crystal violet staining of adhered biomass. However, biofilm formation seemed to be subtly affected in a strain lacking the entire *eps2* region^36^.

**Figure 1:**
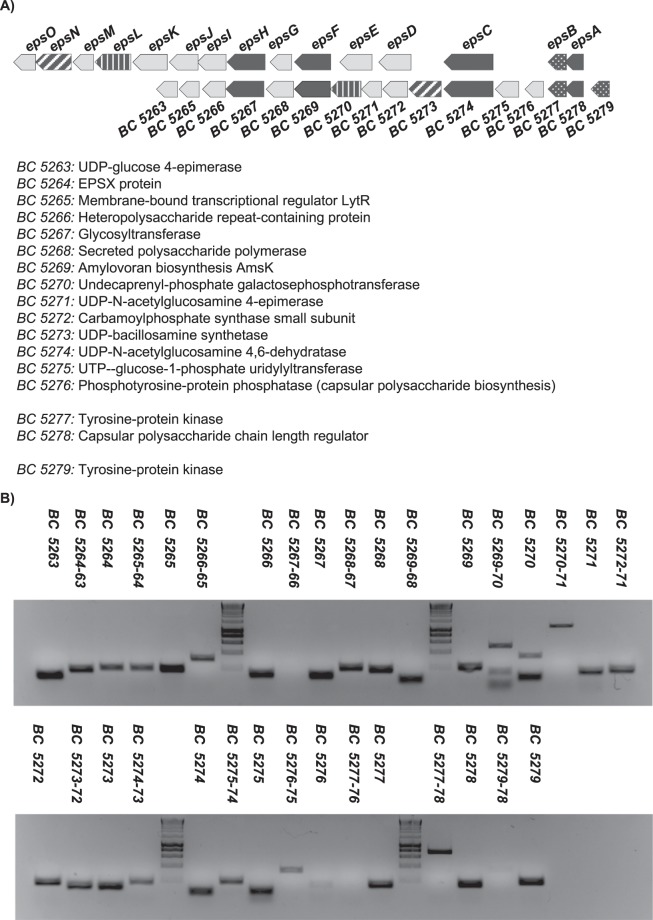
The *eps1* region is organized in multiple transcriptional units. (**A**) Comparison of the genetic structures of the *B. cereus eps1* region and the *B. subtilis epsA-O* operon. Conserved genes are in black and weave and unique genes are marked in grey. Top, a gene cluster predicted to be involved in the production of an EPS in *B. cereus*. Bottom, the operon involved in the synthesis of the biofilm EPS in *B. subtilis*. Automatic annotation of the genes in the *eps1* region is shown. (**B**) qPCR with cDNA obtained by reverse transcribing RNA isolated from a broth culture of *B. cereus* grown for 24 h at 30 °C.

Before initiating specific studies designed to determine the functions of each region, we first studied their genetic organization. Before performing any experiment, we confirmed by PCR analysis the genotype of the *eps* mutants using specific pair of primers (Table S2 and Fig. Suppl. 1B). The *B. cereus eps1* region was compared with the *B. subtilis epsA-O* region to confirm previously reported similarities^35^. Comparison of the genetic regions showed poor homology, with species-specific genes and duplications. Therefore, the different operon arrangement might explain the lack of a phenotype in a knock-out mutant strain (Fig. 1A). To determine if this region is organized as a single operon, we performed RT-PCR with total RNA isolated from a liquid culture of *B. cereus* grown at 30 °C for 24 h (Fig. 1B). The results indicated that the *eps1* region appears to consist of three transcriptional units and an orphan tyrosine-protein kinase: (i) *BC5279*, (ii) *BC5278-BC5277*, (iii) *BC5276-BC5267*, and (iv) *BC5266-BC5263*. An *in silico* analysis of this region also predicted three putative promoter regions at the beginning of each of the transcriptional units.

**Figure 2:**
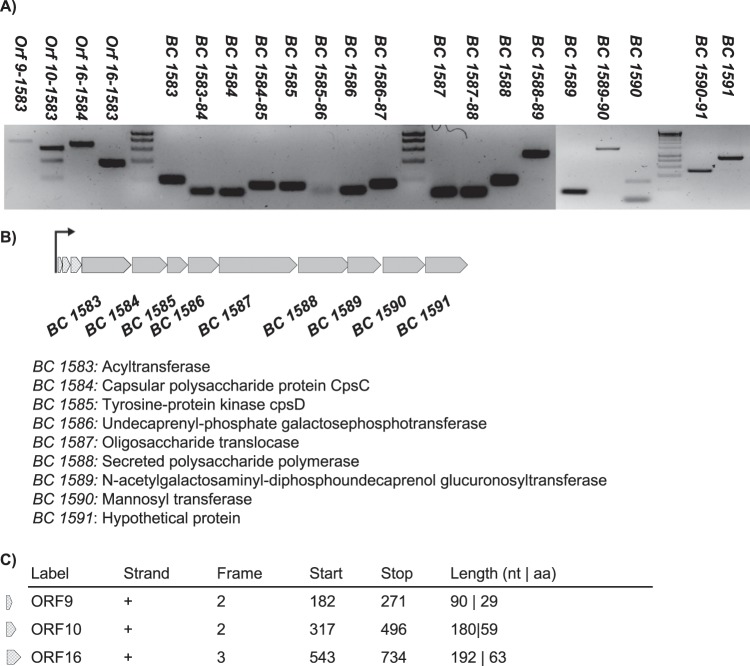
The *eps2* region is organized in a single transcriptional unit. (**A**) qPCR with cDNA obtained by reverse transcribing RNA isolated from a broth culture of *B. cereus* grown for 24 h at 30 °C. The arrow indicates the hypothetical promoter region of the operon. (**B**) The genetic structure determined from the RT-PCR results and the automatic annotation of the genes included in the operon. (**C**) Upstream putative ORFs found via ORF-Finder analysis and included as an integrant of the operon.

The same analysis performed on the *eps2* region revealed that the nine genes are organized in a single transcriptional unit (Fig. 2). To confirm that any additional genes were excluded from the operon, we performed further *in silico* analysis of the 843 base pairs of the intergenic region upstream of the *BC1583* locus using the software ORFfinder. RT-PCR analysis confirmed that the poly-cistronic mRNA also includes the three putative ORFs; however, these additional ORFs were excluded from the knock-out mutation of this genomic region, which did not affect to the loss-of-function phenotype of the *eps2* mutant strain^36^.

### The *eps2* region, but not the *eps1* region, is linked to biofilm formation

**Figure 3:**
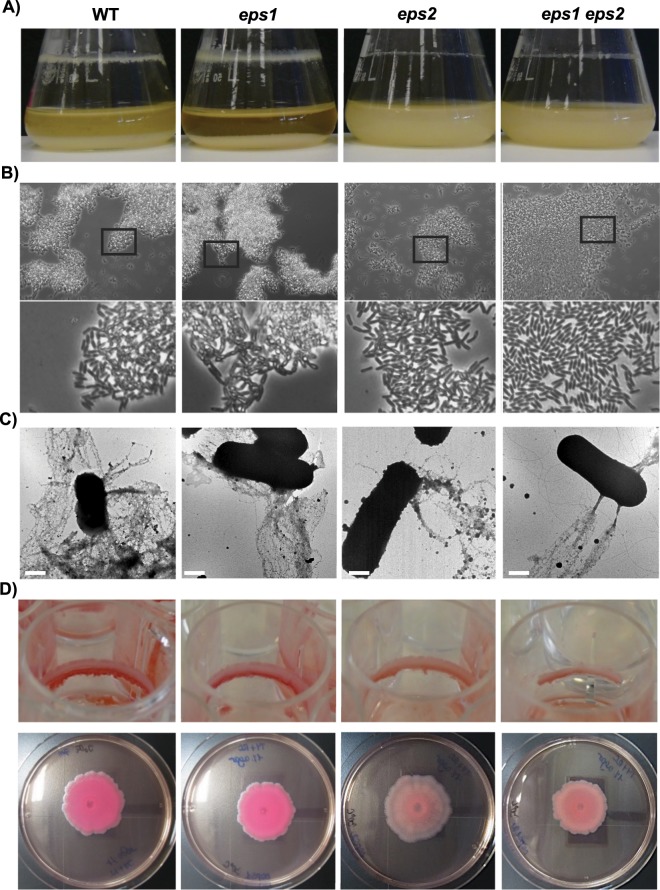
Biofilm phenotypes of the WT and *eps-*mutant strains. (**A**) Liquid cultures in TY medium incubated for 24 h at 30 °C with 150 rpm shaking showed that the WT strain yielded the largest amount of biomass attached to the walls of the flask compared to the strain mutated in the *eps2* region. (**B**) Phase contrast micrographs of the spent medium from liquid cultures show the presence of bacterial clumps in the media from the WT and *Δeps1* strains. The bottom pictures are closer views of the squared areas in the original pictures (**C**) Transmission electron micrographs of negatively stained cells decorated with fibers reactive to anti-TasA antibodies. (**D**) Congo Red biofilm assays in: Top, TY liquid medium supplemented with Congo Red and incubated without shaking at 30 °C for 6 days, and bottom, TY medium solidified with 1.5% agar supplemented with Congo Red and incubated at 30 °C for 72 h. The bars in D = 1 μm.

Previous data led us to propose that *eps2* makes a subtle contribution to biofilm formation, as revealed by crystal violet staining of biomass adhered to an abiotic surface. However, we wanted to decipher the real implications of these two regions in biofilm formation, either alone or in combination. Therefore, we constructed a *Δeps1 Δeps2* double mutant strain (Fig. Suppl. 1B) to search for collaborative functions. Biofilm assays in shaken liquid cultures of the *Δeps*2 strain showed clear defects in the quantity, consistency and continuity of the air-liquid interphase biofilm ring adhered to the glass (Fig. 3A). Reflecting the differences in the expression patterns of *eps1* in biofilm and planktonic cells (Fig. Suppl. 1), the *Δeps1* strain was not impaired in biofilm formation compared to the wild type (WT) strain, and the *Δeps1, Δeps2* double-mutant strain showed the same phenotype as the *Δeps2* single mutant strain (Fig. 3A). In parallel to evaluating the ring-adhered biomass, we also observed significant bacterial cell sedimentation in cultures of the WT and *Δeps1* strains (Fig. 3A). Consistently, imaging of aliquots of the supernatants from cultures of the WT and *Δeps1* strains with a phase contrast microscope revealed bacterial clumps that were absent in spent medium from the *Δeps2* single-mutant strain and from the *Δeps1, Δeps2* double-mutant strain (Fig. 3B). In a previous study^37^, we showed the contribution made by the TasA protein to adhesion to abiotic surfaces; therefore, we wondered whether the reduced adhesion phenotype of the *Δeps2* mutant strain might be caused by the absence of this protein. First, we observed no significant changes in the *tasA* expression levels between the *Δeps1* or *Δeps2* mutant strains and the WT strain in RT-PCR analyses of RNA extracted from biofilm-associated cells (Fig. Suppl. 2). Second, immunogold labeling with anti-TasA antibodies and subsequent electron microscopy imaging revealed the presence of similar amounts of TasA fibers decorating the surfaces of the WT, *Δeps1* and *Δeps2* cells, with no significant differences in their appearance (Fig. 3C). Based on these data, we excluded the possibility that the reduced adhesion of the *Δeps2* strain to abiotic surfaces was due to the absence of TasA or its inability to assemble fibers.

Prompted by the well-known staining properties of certain EPSs, we performed biofilm assays on agar plates and in static liquid cultures in the presence of Congo Red, a dye commonly used to reveal the presence of certain types of EPS (Fig. 3D)^13,38,39^. As previously reported^36^, the *Δeps1* single mutant cells formed a biomass on the walls of the wells in static cultures, similar to that formed by the WT cells; however, subtle differences could be appreciated in the *Δeps2* strain, and major defects were observed in the double-mutant strain. These assays also clearly defined a red zone visible in the bottom part of the adhered biomass of the WT and *Δeps1* strains that was absent in the biomass of the *Δeps2* single- and *Δeps1 Δeps2* double-mutant strains (Fig. 3D, top). Complementary to these findings, two distinct phenotypes emerged from the same experiments performed instead on agar plates: the absence of reddish staining and robust spreading of colonies of the *Δeps2* single- and *Δeps1 Δeps2* double-mutant strains (Fig. 3D, bottom). Taken together, these findings support two primary conclusions: (i) The two EPSs have divergent functions, i.e., while EPS2 seems to be relevant for establishing the sedentary lifestyle associated with biofilm formation, EPS1 appears to be more important for colony mobility; and (ii) the two putative EPS species likely have different chemical compositions, as indicated by the binding of EPS2, but not EPS1, to Congo Red. To evaluate differences at chemical level, EPSs from these strains were cold-ethanol precipitated, separated in size exclusion chromatography (SEC) and fractions subjected to the phenol-sulfuric acid method^40^ (Fig. Suppl. 3A–D, insets). Fractions that firstly eluted in the SEC column, and thus attributed to larger polysaccharide structures, were initially analyzed in Attenuated total reflectance-Fourier transform infrared spectroscopy (ATR-FTIR) to confirm the presence of functional groups characteristics of EPS^41^ (Fig. Supp. 3E). As previously reported, in the range of 3600-3200 cm^−1^ the samples exhibit a broad peak for the O-H stretching vibration of the polysaccharide^42^. The two weak bands between 2930-2850 cm^−1^ are assigned to C-H stretching vibrations (CH_2_ and CH_3_ groups). Peaks at 2360 cm^−1^ and 1658 cm^−1^ might be respectively attributed to NH stretching and C=O stretching vibration of N-acetyl group that might be protecting OH groups. The symmetrical deformations of CH_2_ and C-OH deformations are weak bands that appear between 1440-1460 cm^−1^. For polysaccharide identification, the strong peak in the range 1200-1000 cm^−1^ is caused by C-O-C and C-O groups in pyranose ring^43^. The coexistence of α and β anomeric form is confirmed by the presence of bands at 860 and 915 cm^−1^. These fractions were next chemically studied by hydrolysis and subsequent derivatization for further separation in gas chromatography and mass spectrometry. The major component monosaccharides of all extracts were found to be glucose, galactose and mannose together with other minor components like ribose, rhamnose or xylose, indicating that the EPS produced is a heteropolysaccharide (Figs. Suppl. 3A–D and Suppl. 4). Interestingly, purified samples of the *Δeps2* mutant strains exhibit fucose units in the analysis. Based on this findings, one can assume that these differences at sugar composition might reflect the divergent behavior in binding devices features of each strain.

### The products of the *eps1* and *eps2* genomic regions are not directly involved in cellular auto-aggregation

**Figure 4:**
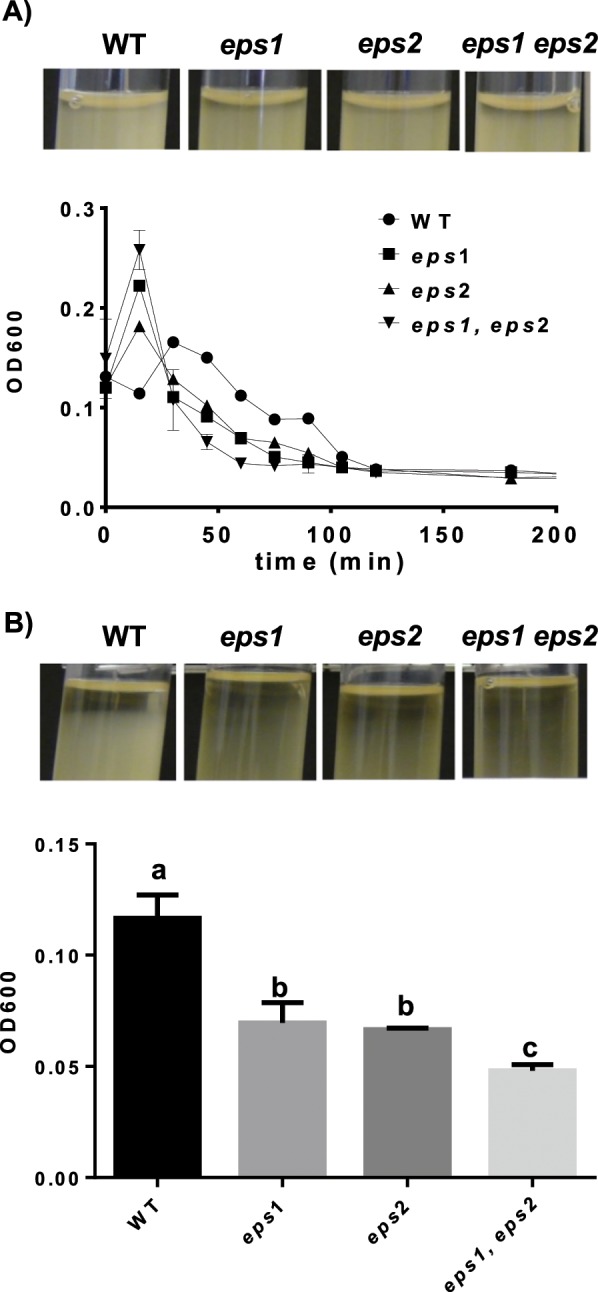
Eps1 and Eps2 prevent cellular auto-aggregation. The optical densities (ODs) of cultures grown in TY medium incubated at 30 °C for 16 h with shaking at 150 rpm were adjusted to OD = 3 before the experiments were initiated. (**A**) Top, representative images of the tubes after 5 h of incubation; bottom, OD values of the air-liquid interface in the cell suspensions under static conditions at room temperature. (**B**) Top, representative images of the cultures after 9 h of incubation; bottom, OD values of the liquid cultures after 9 h of incubation, with sampling at the height of the grade bar. Means compared using t-test (P < 0.05).

EPSs are known to possess adhesive properties that promote cell-to-cell interactions, an early step in the formation and maturation of bacterial biofilms^44^. The observation of bacterial clumps in the spent media from agitated cultures of the WT and *Δeps1* strains led us to consider the importance of EPS2 in cell aggregation (Fig. 1). To definitively address this phenotype, we performed auto-aggregation assays using two complementary methods: (i) recording time-dependent variations in the optical density (OD) of the upper part of liquid cultures^45^, and (ii) measuring the OD at a defined depth in liquid cultures at the end of the experiment^46^. To perform these assays, bacterial cells were grown overnight at 28 °C, and then, after adjusting the culture ODs to a uniform value, cell suspensions were incubated at room temperature (RT) with no agitation. Samples were taken every 15 min for the first method (Fig. 4A), and for the second method, the ODs were measured at the same depth in all of the cell suspensions after 9 h of incubation (Fig. 4B). Our findings indicated that absence of either of the *eps* regions led to faster aggregation kinetics compared with that of the WT strain, and this effect was amplified in the *Δeps1 Δeps2* double-mutant strain during the first 5 h of stationary incubation (Fig. 4A). Consistently, the OD values obtained with the second method were lower in the mutant strains compared with that of the WT strain after 9 h of incubation (Fig. 4B). Furthermore, after 24 h of incubation, sediments were visible in the bottoms of the tubes in the cultures of the mutant strains, but not in that of the WT strain (Fig. Suppl. 5). These results suggested that *eps1* and *eps2* are irrelevant for cellular auto-aggregation and indicated that other cell surface factors (possibly hidden by the presence of EPS) might be directly involved in this cellular process. Thus, in the absence of either EPS species, the cells interact more efficiently and increase their sedimentation rate, suggesting a mechanism through which EPS could indirectly control cell auto-aggregation. We next hypothesized that either TasA or CalY (extracellular proteins involved in *B. cereus* biofilm formation) might function with the EPS to regulate bacterial auto-aggregation at the structural level. Strains mutant for either *tasA* or *calY* aggregated similarly to the WT strain or to either of the *eps* mutant strains (Fig. Suppl. 6). The *Δeps1*, *eps2*, *tasA* triple-mutant strain showed aggregation kinetics similar to those of the WT strain; in contrast with the phenotype of the *Δeps1*, *eps2*, *calY* triple-mutant strain that was severely defective in cellular auto-aggregation. These findings supported the functionality of CalY in bacterial cell-to-cell interaction and demonstrated that CalY is a bacterial factor involved in auto-aggregation whose functionality may be hidden or modulated by the presence of EPS (although other such factors may also exist).

### The *eps1* and *eps2* regions antagonistically influence cell motility

**Figure 5:**
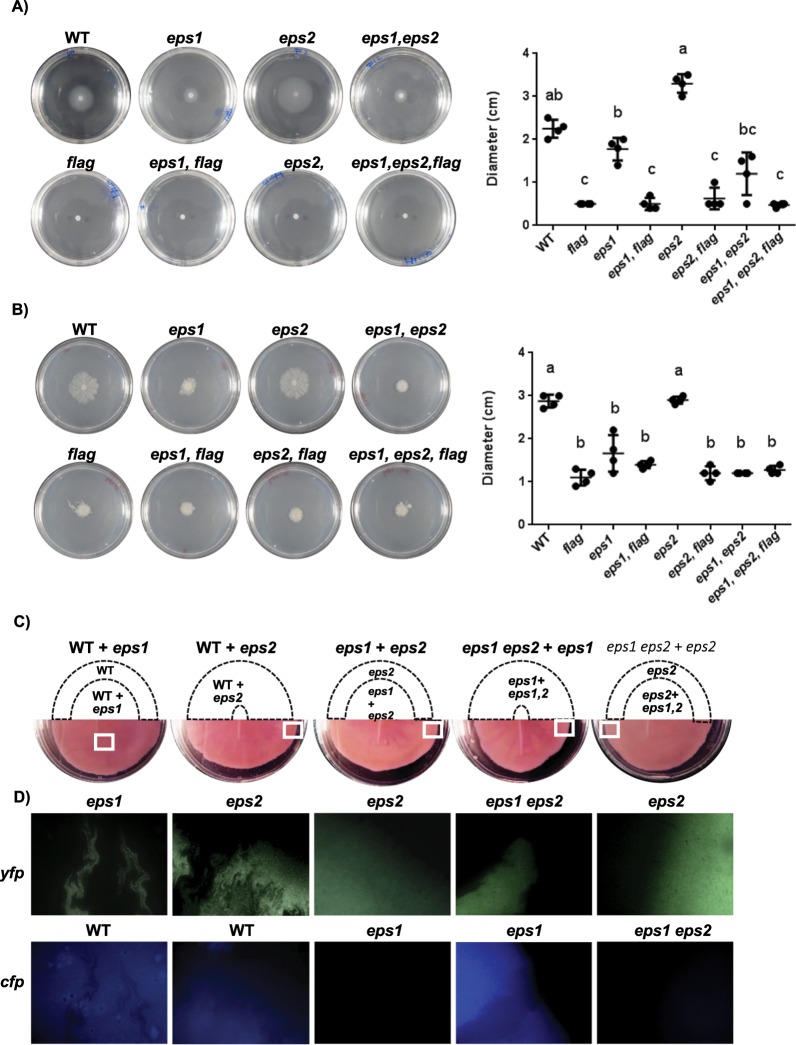
EPS1 and EPS2 contribute differently to bacterial cell motility. Cell suspensions were spotted in the centers of the plates, which were then incubated at 28 °C before examining the motility. (**A**) Images taken from the swimming agar plates (0.3% TY agar plates, 24 h). (**B**) Images taken from the swarming agar plates (0.7% TY agar plates, 72 h). The graphs represent the values of the colony diameters at the end of each experiment. A Tukey statistical analysis was realized (p-value < 0.01) and the significant differences are indicated with letters. (**C**,**D)** Bacterial strains constitutively expressing YFP or CFP were mixed at a 1:1 ratio and then spotted in the centers of the plates. (**C**) The motility and Congo Red binding phenotypes were analyzed on TY swarming agar plates supplemented with Congo Red. The drawing indicates the zone occupied by each strain in the corresponding complementation experiment and marked with squares in the original picture (bottom). (**D**) Fluorescence microscopy images showing the presence of each bacterial strain in the same section indicated in panel C, bottom.

Several EPSs have been reported to play roles in motility in different bacterial species, and we noticed differences in the colony sizes of the *eps* mutant strains grown on typical solid agar plates (Fig. 3D, bottom). To determine if EPS1 or EPS2 is involved in bacterial motility, we seeded suspensions of WT or mutant cells on 0.3% or 0.7% agar plates to assay swimming or swarming motility, respectively (Fig. 5A,B). The *Δeps2* cells swam more efficiently than did the WT or *Δeps1* cells, and we observed a reversion to the *eps1* phenotype of the double mutant *eps1, eps2* (Fig. 5A). This result suggested that EPS2 has a negative effect on swimming and was consistent with the propensity for cellular clumping observed in liquid cultures 24 h post-inoculation (Fig. 3A). On swarming agar plates, the *Δeps1* and double-mutant colonies spread less than the WT colonies, and *eps2* deletion did not result in any noticeable change in colony spreading (Fig. 5B). Taken together, these findings indicate that EPS2 likely antagonizes cell motility, while EPS1 seems to promote social movement. To really decipher the involvement of EPS1 in motility different from swimming and swarming, we did mutate the *flag* allele in all the strains. No significant differences were observed in colony mobility, showing no swimming or swarming motility of all strains lacking the *flag* allele, confirming that EPS1 is involved in motility dependent on flagella and that the previous results are not a result of a different type of mobility (Fig. 5A,B, bottom).

Based on these findings, we reasoned that mixing cells from each strain might restore the WT phenotype. To test this hypothesis, we performed strain complementation experiments on swarming agar plates supplemented with Congo Red and tested for reversion of the swarming motility and dye staining phenotypes (Fig. 5C). When both tested strains possessed the *eps2* region, the colony color was homogeneously reddish. When one of the strains lacked the *eps2* region, colored sectors appeared. In colonies in which neither or both strains possessed *eps1*, the sectors reached the colony border; however, these sectors decayed from the center to the border of the colony when only one strain lacked *eps1* (Fig. 5C). These results support our view that EPS1 is necessary for robust social motility, given that the strains lacking *eps1* showed delayed movement and remained in the colony centers. Two strain mixtures, WT + *Δeps1* (reddish) and *Δeps2 + Δeps1Δeps2* (pinkish) showed no sectors, given that the color phenotypes of both strains were similar. To clearly visualize the cellular distribution of each strain in the complete community, these strains were transformed with replicative plasmids constitutively expressing either *yfp* or *cfp* (Fig. 5D). Fluorescent microscopy imaging confirmed the formation of sectors in the mixtures and supported the previously-stated hypothesis regarding the positive contribution of EPS1 to social movement on swarming agar plates. A mixture of WT and *Δeps1* cells showed reduced fitness in the cells lacking *eps1*, which remained close to the original spot area, and the colony borders were mainly dominated by WT cells (Fig. 5D). Similarly, the *Δeps1 Δeps2* double-mutant cells were less competitive when combined with *Δeps2* cells. These results suggest that only cells producing EPS1 show enhanced movement; thus, the function of EPS1 in motility is likely on the level of individual cells, not shared with the neighbors, rather on the level of the entire community, the latter of which has been reported for the secretion of surfactants and other EPSs that can facilitate the motility of non-producer cells^47^.

### EPS2 is more relevant for the adhesion of *B. cereus* cells to human epithelial cells and the zebrafish gut

**Figure 6:**
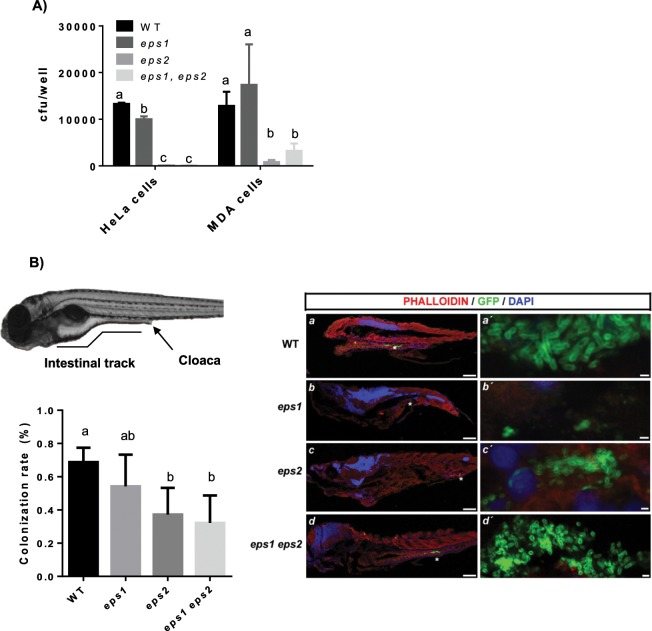
EPS2 is more important for the adhesion of *B. cereus* cells to human epithelial cells and to the zebrafish gut. (**A**) Adhesion assays in cultures of the human HeLa and MDA epithelial cell lines. Means compared using t-student (P < 0.05). (**B**) Top left, representative image of a zebrafish larva 6 days post-fertilization (dpf). Bottom left graph, rates (±SD) of 6-dpf zebrafish larvae harboring fluorescent bacteria in the GI tract 24 h after feeding with the WT, *Δeps1, Δeps2* or double-mutant *B. cereus* strains (1 E8 ucf/ml). Means compared using t-student (P < 0.05). Right panel, bacterial localization in the gut of the zebrafish larvae after infection. The bacterial strains were transformed with a plasmid expressing the *yfp* gene under the control of a constitutive promoter. a-d´: Fluorescent Z-stack confocal microscopy images (the bacteria marked with asterisks in a-d are magnified in a´-d´). Scale bars = 200 μm in a,b,c,d, 100 μm in a-d, and 1 μm in a´-d´.

The previous *in vitro* experiments suggested a major contribution of *eps2* to the adhesion to abiotic surfaces and to biofilm formation. Thus, we wondered if these findings could be extended to biotic surfaces. In light of the pathological implications of *B. cereus* in humans, we initially tested the adhesion abilities of these strains to HeLa and MDA epithelial cells as a model for human infection. As expected, based on the results of the *in vitro* experiments, the *Δeps2* strain was severely impaired in adhesion, while the *Δeps1* strain behaved like the WT strain. Like the *Δ*eps2 strain, the double-mutant strain showed reduced adhesion to both epithelial cell lines (Fig. 6A).

After confirming the adhesive properties of EPS2 to epithelial cells, we evaluated the implication of this EPS in bacterial adhesion to a complex biotic surface, the zebrafish (*Danio rerio*) intestine, a model that anatomically mimics the conditions of the mammalian gut due to the presence of finger-like protrusions (villi) on the intestinal epithelium and to the presence of a diverse cell population, including immune cells^48^. After feeding zebrafish larvae with WT, single- or double-mutant bacteria harboring a reporter plasmid that constitutively expressed YFP, the presence of the bacteria in the fish gut was evaluated by confocal fluorescence microscopy (Fig. Suppl. 7). Examination of fish showed that 24 h post-feeding, 67% of the larvae retained fluorescent bacteria in their gut. Larvae fed with *Δeps1, Δeps2* or double-mutant cells showed decreased retention rates of 54%, 37% and 32%, respectively. Based on these differences in the dynamics of the bacterial populations, we expected different distribution patterns of the cells in the intestinal tract. A detailed examination by confocal microscopy using triple labelling with phalloidin to stain the epithelial cells, DAPI to stain the nuclei, and anti-GFP, confirmed the presence of bacterial cells of each specific strain in different regions of the intestinal tract (labeled with asterisks) (Fig. 6Ba–d, right panel). Representative images of zebrafish intestines showed that more WT, *Δeps2* and double-mutant (*Δeps1, Δeps2*) cells accumulated in the mid- and posterior-gut compared with the amount of *Δeps1* cells in the same regions.

The experiments with the epithelial cell lines (37 °C) and zebrafish (RT) were performed at different temperatures; thus, we asked if different culture temperatures could lead to different expression levels of the two EPSs, thus altering their functionality. An RT-PCR analysis revealed that both *eps* regions were more efficiently expressed at 30 °C than at 37 °C, especially *eps2*, which showed a nearly 5-fold higher expression level at 30 °C (Fig. Suppl. 8). A comparison of the changes in the *eps1* and *eps2* expression levels indicated that *eps1* expression is less sensitive to temperature changes. Thus, the lower importance of EPS1 in the adhesion to biotic surfaces could reflect the chemical differences between EPS1 and EPS2 rather than differences in their expression levels.

## Discussion

Bacteria normally possess a repertoire of genetic regions devoted to the synthesis of different polysaccharides^22^. These polymers can play various structural functions, including coating of the cell wall, capsule formation, spore surface decoration, and providing adhesion or structural support for biofilms; furthermore, they also offer protection against various stresses, provide a storage reservoir for carbon and protect the bacterial cells against host defenses^49,50^. *B. cereus* has been shown to produce several different polysaccharides, including (i) exopolysaccharides associated with sporulation (genes *BC0484-90)*^51^, (ii) two spore-decorating sugars (genes *BC3358-61*)^31^, and (iii) two secondary cell wall polysaccharides (SCWP) (genes unknown)^52^. In this work, we have investigated the roles of two *B. cereus* genomic regions putatively dedicated to the production of two different polysaccharides, referred to as *eps1* and *eps2*. The data obtained from our study indicate that the two polysaccharides play different, but complementary, roles in *B. cereus* multicellularity, with EPS1 mainly contributing to motility and EPS2 mainly supporting adhesion, biofilm maturation and interaction with host cells.

One of the cellular processes related to biofilm formation is bacterial cell auto-aggregation^44^. The data from our auto-aggregation assays showed that the lack of either *eps* region resulted in increased sedimentation rates, suggesting that the EPS may mask bacterial surface structures responsible for cell-cell interactions, a phenomena also reported in other bacterial species^6,53^. This finding was, however, contradictory to the cellular aggregation observed in flasks, a discrepancy that is due to the different timing of both experiments resulting in different ages of the cultures and, consequently, the physiological states of the cells, given that the auto-aggregation assays were performed after 16 h of incubation, before the formation of a visible biofilm ring, and the flask aggregation assays were performed after 24 h of incubation with vigorous shaking and after well-developed biofilm rings were visible. Therefore, these findings should be considered complementary: one set shows the auto-aggregation properties of planktonic cells, while the other set shows the aggregation in mature cultures, in which the bacteria have entered a physiological state in which they are particularly prone to form biofilms. Our results also suggest that additional elements beyond the EPSs might be involved in the cellular attractive and repulsive forces regulated in response to culture conditions and responsible for the cellular auto-aggregation and cell-cell interactions^54^. For instance, in *B. thuringiensis*, the S-layer is released at the onset of stationary phase, changing the surface characteristics of the bacteria^55^. The observation that the cellular auto-aggregation of the *eps1, eps2, calY* triple-mutant strain was affected suggests a direct role for CalY in this process and that the regulation of this cellular process depends on interactions between different structural components. Nevertheless, the biofilm assays performed in shaking flasks clearly showed that the loss of *eps2* resulted in reduced aggregation and reduced biofilm formation, consistent with previous works that demonstrated that auto-aggregation is directly linked to biofilm formation^44^.

Motility is an important bacterial feature that contributes to adaptation, interaction, survival and biofilm formation^56–58^. In addition to the single-cell movement produced by flagella, bacteria have evolved sophisticated social movements in which certain molecules produced by a sub-population of cells are used to facilitate the movement of the entire bacterial community^59^. Consistent with the implication of EPS2 in the development of a sessile lifestyle, the results obtained from our motility assays indicated a negative effect of EPS2 on swimming motility, probably because of the adhesive properties that mediate cell-cell interactions and lead to the formation of cell clumps^5^. Contrary to this finding, EPS1 seemed to be more important for the development of efficient movement. Indeed, complementation experiments in which two strains were co-seeded revealed reduced fitness in *eps1* mutant cells placed in competition with the other strains, i.e., the movement of these cells was consistently delayed and they were ultimately restricted to the colony inner areas. The difference in the functions of EPS1 and EPS2 can be considered positive in terms of the need for *B. cereus* to adapt to the variety of environmental conditions that it may encounter during its life cycle, i.e., (i) promoting motility for the efficient colonization of highly competitive habitats, such as vegetable tissue or mammal and insect guts^17^, or (ii) switching to a sessile lifestyle in less competitive, but more harsh habitats, such as on the surfaces of industrial or medical devices^60^. The observation that the *eps1* mutant is impaired, but not entirely deprived of motility on swarming agar plates suggests that the products of the *eps1* region must be involved in social, collective movement, but not directly in swarming motility^61^. In *B. subtilis*, swarming is not affected by the deletion of the *eps* operon, while flagellum independent sliding depends on the produced EPS^62^. Our results in *B. cereus* suggest the requirement of both flagella and EPS1 for efficient surface colonization. This is reminiscence to the mechanism required for the wandering colonies of *Paenibacillus* that depends on functional flagella and the secreted protein CmoA, speculated to be a water recruiting protein^63^. *B. cereus* EPS1 might have similar function to surfactin swarming in *B. subtilis*, with the particularity that it is not shared for the community.

We observed that the *eps1* expression level is less sensitive to temperature variation, while the *eps2* expression level is very sensitive to temperature shifts, becoming downregulated at 37 °C. In mammalian niches, *B. cereus* cells are more likely to attack the host cells, remaining in a planktonic state^36,64^. Unless other factors influence *eps2* expression, in a scenario in which *B. cereus* is predisposed to attack its mammalian host, small amounts of EPS2 could support efficient adhesion of the *B. cereus* cells to the host’s epithelial cells. Our results in the zebrafish gut are consistent with the results of our *in vitro* adhesion assays and support the contribution of EPS2 to *B. cereus* persistence in a natural environment. However, the differences in adhesion were less evident *in vivo*, probably due to the higher complexity of the gut environment, where a mucus layer covers the lumen and other resident microorganisms may interact with *B. cereus*^48^.

In summary, our results reveal that *eps2* is involved in adhesion, biofilm formation, cell-cell cohesion and host interaction, while *eps1* might play a role in promoting a kind of social motility. The *eps1* region shows similarities with the *B. subtilis epsA-O* operon, which encodes the factors required for the synthesis of the structural biofilm EPSs. Some previous work has demonstrated that this EPS stimulates anti-inflammatory M2 macrophages, inhibiting T cell activation^65^. Thus, we tentatively propose that the *B. cereus eps1* region might have evolved to exert enhanced immune modulatory effects, while losing most of its structural functions; however, this suggestion requires further investigation.

## Methods and Materials

### Bacterial strains and culture conditions

The bacteria used in this study are listed in Annex Table S1. Bacteria were routinely grown in TY broth (1% tryptone, OXOID), 0.5% yeast extract (OXOID), 0.5% NaCl, 10 mM MgSO_4_, and 0.1 mM MnSO_4_). Biofilm assays were performed in TY both or on the same medium solidified with 1.5% agar^37^.

### Construction of *B. cereus* mutants

*B. cereus* mutants were obtained by electroporation using the plasmid pMAD^66^. The primers used to generate the mutagenesis constructs are listed in Annex Table S2. The constructs were created by joining PCR. In the first step, regions flanking the target genes were amplified separately, purified, and used for the joining PCRs. These PCR products were digested and cloned into the pMAD vector digested with the same enzymes. The resulting suicide plasmids were then used to transform *B. cereus* electrocompetent cells as described previously, with some modifications^37^. Electroporation was performed with 10 µg of plasmid in 100 µL of electrocompetent *B. cereus* cells in 0.2-cm cuvettes using the following electroporation parameters: voltage 1400 kV, capacitance 25 µF, resistance 400 Ω. After electroporation, the cells were incubated in LB for 5 h and then seeded on LB plates supplemented with X-Gal and erythromycin, followed by incubation for 72 h at 30 °C. Blue colonies were selected and streaked to trigger allele replacement. Finally, white, MLS-sensitive colonies were selected, and deletion of the target gene was verified by colony PCR analysis and sequencing of the amplicons.

### Plasmids for fluorescence microscopy analysis

The plasmid pHCMC02 was slightly modified to constitutively express a fluorescent protein (either YFP or CFP)^67^. The original promoter in the plasmid was replaced by the constitutively expressed promoter of the *upp* gene from *B. cereus* using the primers listed in Annex Table S3. The WT strain and the *Δeps1, Δeps2* and double-mutant strains were transformed with the constructed vectors, and positive clones were isolated and checked for suitability. The images were collected using a Leica SP5 HyD MP Multiphoton/Confocal Microscope equipped with a tunable titanium-sapphire infrared laser capable of emitting between 700 and 1040 nm.

### Biofilm assays

The *B. cereus* biofilm assays were performed by assessing the bacterial adhesion to abiotic surfaces in 24-well plates and 250 mL flasks^68^. The cultures were grown in liquid TY medium at 30 °C with or without agitation. To visualize the population dynamics in the biofilm, the well adhered biomass (24-well plates) of each strain was recovered at different time points and resuspended in TY. The optical density at 600 nm was measured in a spectrophotometer and total number of viable cell bacteria was estimated and expressed as CFU/mL.

The Congo Red (CR) staining of the *B. cereus* colonies was performed as described above but using TY agar medium supplemented with CR and Coomassie Brilliant Blue G at final concentrations of 20 µg/ml and 10 µg/ml, respectively; the dyes were filtered and added to sterilized TY medium^69^.

### Auto-aggregation assays

Flasks containing 25 ml of TY medium were inoculated with *B. cereus* and incubated with agitation at 200 rpm at 28 °C overnight. The cultures were divided into two fractions. The OD_600_ values of the 10 ml culture aliquots for each strain were adjusted to 3 using the supernatant obtained from centrifugation of the other fraction. Auto-aggregation was measured using a final point measurement^44^ and a kinetic method^70^.The tubes were then incubated vertically at RT without agitation or movement. At each sampling time, 10 µL aliquots were carefully taken from the air-liquid interface of the medium and diluted in 90 µL of TY medium to measure the OD_600_ values in a plate reader (Omega).

### Swarming and swimming motility assays

Petri dishes with TY medium containing 0.7% agar (for swarming) and 0.3% agar (for swimming) and 20 µg/ml of CR were used to assess colony expansion^71^. Bacterial cell suspensions at OD_600_ = 1 were seeded in the centers of the plates in 5-µl drops. The plates were then incubated at 28 °C for 24, 48 and 72 h. The colony diameters were measured, and the data were statistically treated to obtain the means and SDs, n = 6–9.

### RT-PCR and qRT-PCR

Total RNA was isolated following the protocol previously described^36^. The quality of the RNA extraction was tested by spectrometry using NanoVue GE equipment and electrophoresis in agarose gel. cDNA was obtained from the total RNA samples using the Titan One RT-PCR System (Roche). To determine the mRNA levels of the products expressed from the operons containing the *eps1* and *eps2* regions, PCRs were performed using the cDNA as a template and the primers listed in Annex Table S4. Positive controls for each primer pair were included using genomic DNA as the template, and for negative controls, the original RNA extraction was used as a template to ensure that the extracted RNA was not contaminated with genomic DNA.

For the quantitative real-time PCR, we measured the transcript levels of the *eps1* (*Bc5274*) and *eps2* (*Bc1583*) sequences at 28 and 37 °C using qRT-PCR. The reaction was performed using the Power SYBR Green Master Mix (Bio-RP) following the manufacturer’s recommendations. The reactions were performed in triplicate in 96-well plates in a reaction volume of 20 μL containing 1 μL of cDNA, 10 μL of SYBR Green Master Mix, 0.6 μL of a 10 nM solution of each primer, and 7.8 μL of ddH_2_O. The reaction was started with an initial denaturation at 95 °C for 3 min followed by 40 cycles of amplification: 95 °C for 20 s, 56 °C for 20 s and 72 °C for 30 s. The statistical significance was assessed by repeated measures ANOVA with post-hoc paired Student’s t test. Results with p < 0.05 were considered statistically significant.

### Immunocytochemistry and transmission electron microscopy

Biofilm cells were harvested 48 h post-inoculation and gently resuspended in 1 ml of phosphate-buffered saline (PBS). Carbon-coated grids were placed on top of 20-μl samples for 1 h. The grid was washed in PBS for 5 min, fixed with a solution of 2% paraformaldehyde for 10 min, washed in PBS and blocked with bovine serum albumin (BSA)-PBS for 30 min. The monoclonal anti-TasA primary antibody was raised against a peptide (KGISAGKSDKFK) from the TasA sequence, which is different from that used by Caly (NovoPro). The anti-TasA antibody was used at a dilution of 1:50 in 2% BSA-PBS for 1 h at RT. The samples were washed three times with TBS (50 mM Tris-HCl, 150 mM NaCl, pH 7.5) -Tween20 (0.1%) for 5 min. The grids were then exposed to the secondary goat antirabbit antibody conjugated to 10-nm gold nanoparticles at a dilution 1:1000 for 1 h. The samples were then washed two times with TBS-Tween20 and in a drop of water for 5 min. Next, the grids were treated with 2% glutaraldehyde for 10 min, washed in water for 5 min, and then negatively stained with a 1% uranyl acetate solution. The samples were dried and examined using a FEI Tecnai G2 20 TWIN transmission electron microscope.

The immunofluorescence analysis was performed by blocking non-specific binding sites with 16% sheep serum, 1% BSA and 0.5% Triton X-100 in PBS (SBT) and incubating the tissue slides overnight at 4 °C in the primary antibody (GFP, 1:250 dilution). The samples were subsequently washed in PBS (3 × 5 min), incubated (2 h) in the proper FITC-conjugated secondary antibody (anti-chicken IgG, Jackson, 1:100 dilution) + Alexa Fluor 647 Phalloidin (Life technologies, 1:200 dilution) + DAPI (1:2000 dilution) and washed in PBS (3 × 5 min). All of the samples were mounted in a 1:1 PBS/glycerol solution and analyzed under a SP5 laser confocal microscope (LEICA).

### EPS extraction

Exopolysaccharides substances were extracted following procedures previously published with modifications^73^. *B. cereus* WT and related mutants in the different EPS were growth overnight in Ty liquid culture and one milliliter was inoculated into 200 ml of fresh Ty liquid medium and incubated with no agitation at 30 °C for 5 days. Cells were harvested by centrifugation at 8000 × g for 10 min and washed with distilled water. After centrifugation, the cell pellets were resuspended in PBS buffer and were mildly sonicated (twice at 30% amplitude for 1 minute) to separate exopolysaccharides from cells. The soluble fractions were stirred on ice and a 100% TCA solution was added in order to precipitate proteins from the EPS extract. The mixture was left at 4 °C overnight and the proteins were removed at 10000 × g for 20 min. Exopolysaccharide from the supernatant were precipitated adding 5 volumes of chilled ethanol under continuous stirring and kept overnight for precipitation at 4 °C. EPS crude was collected by centrifugation (10000 × g, 20 min). The pellets were dialyzed against distilled water overnight (Spectra/Por^®^ dialysis membrane molecular weight cutoff 3.5 kD) and the resulting solution was filtered and applied onto a size exclusion chromatography column (Hiprep^TM^ 16/60 Sephacryl^TM^ S-300HR) eluting with MQ water. Aiming the sugar presence into the fractions, aliquots of each fraction were analyzed by the pheno sulphuric acid method and monitored the absorption at 490 nm in a microplate reader (FLUOstar Omega reader, BMG LabTech). Fractions containing carbohydrates were pooled and lyophilized for further analysis.

### Sugar composition analysis (GC-MS)

Extracted samples (1-2 mg) of EPS were hydrolyzed with 1 mL of HCl/MeOH 3 N (sigma Aldrich) into a 3 ml reacti-vial (Thermo) at 80 °C for 24 h. The mixture was evaporated under N_2_ stream at 50 °C. The residue was washed three times with MeOH and dried again under N_2_ stream. Sililation of hydrolyzed samples were carried out with 300 µl of Tri-Sil reactive (Pierce, Thermo) for 1 h at 80 °C. Excess of the reactive were eliminated by N_2_ stream and 1.5 ml of Hexane was added followed by centrifugation. Recovered supernatant was filtered, dried under N_2_ stream and 150 µl of Hexane (LC-MS grade, Sigma) was added for Gas chromatography analysis. A Zebrón ZB-5 capillary column (30 m × 0.25 mm ID × 0.25 µm df, Phenomenex) was used to separate monosaccharides. Carrier gas flow was set at 1.2 ml/min and injection volume at 1 µl in split mode. The initial column temperature was set at 80 °C for 2 min followed by a temperature gradient of 10 °C/min until 180 °C and a 5 °C/min gradient until 250 °C and then the temperature was kept for 2 min. Ionization in mass spectrometer was performed by electron impact (EI) at 70 eV and ion source temperature at 230 °C. Full scan was set between 50-650 m/z.

### ATR-FTIR

In order to investigate the functional groups in the EPSs samples of *B. cereus* and mutants Attenuated Total Reflection Fourier Transform Infrared spectroscopy (ATR-FTIR) was used. Spectra of solid samples were recorded from 4000 cm^−1^ to 500 cm^−1^ on a Golden Gate Single Reflection Diamond ATR System (Graseby Specac) fitted into a Bruker Vertex 70 FT-IR spectrometer. Standard spectral resolution of 4 cm^−1^ was used and 64 scans were selected for both background and sample measurements. Spectral contributions from residual water vapor were reduced using the atmospheric compensation filter built in the Bruker OPUS software.

### Cell culture adhesion

Bacterial adhesion to human cells cultures was performed using a slightly modified protocol described before^72^. MDA-MB-231 breast adenocarcinoma cells and HeLa cervical cancer cells were obtained from the ATCC and grown in RPMI 1640 medium or DMEM supplemented with glucose (4.5 g/L), respectively, both containing glutamine (2 mM), penicillin (50 IU/mL), streptomycin (50 mg/L), amphotericin (1.25 mg/L), and 10% FBS at 37 °C in a 5% CO_2_ atmosphere. Cells were seeded at 2000 and 1500 cells/well in 96-well plates and incubated for 72 h at 37 °C in a 5% CO_2_ atmosphere to achieve confluence with a cell density of 1·10^4^ cells/well. The medium from the HeLa and MDA cell cultures was replaced with “assay culture” medium (supplemented with glutamine and FBS, without antibiotics or antifungals), and the cells were incubated for 2 h. Next, the culture medium was replaced once again with assay culture medium before the bacterial inoculation. *B. cereus* ATCC14579 and the EPS mutants were streaked on LB agar plates and incubated for 24 h at 28 °C. Tubes containing 5 ml of TY medium were inoculated with *B. cereus* ATCC14579 and the EPS mutants and incubated overnight at 28 °C with vigorous shaking. The bacteria were washed three times with sterile PBS and the OD_600_ values were adjusted to 1 (approx. 10^7^ UCF/ml). Cultures of the human cells in 96-well plates were inoculated with 10 µl of bacterial suspensions at an MOI of 10:1. The plates were centrifuged at 2000 g for 1 min to force the bacteria into contact with the human cells and to avoid bias introduced by motility differences. After incubation for 45 min, the plates were washed 5 times with sterile PBS to remove the non-adhered bacteria. The cells were lysed with 0.1% Triton X-100 for 10 min, and serial dilutions were made from each well and plated on LB agar to titer the number of bacteria that adhered to the cells.

### Danio rerio *gut adhesion assay*

Zebrafish larvae (6 dpf, days post-fertilization) were used for the experiment to assess the proportion of larvae maintaining *B. cereus* cells (introduced into the gut by feeding) 24 h post-feeding. In 50 ml tubes containing 30 ml of E3 medium without disinfectant, 30-40 larvae were fed with bacteria at a final concentration of 1.0e8 UCF/ml (approx. OD_600_ = 1). The larvae were incubated at RT for 12 min. Next, the tubes were cooled on ice until the larvae settled to the bottoms to allow removal of the medium containing excess bacteria (repeated six times). The larvae were then microscopically observed individually, and 10-21 specimens with a visible mass of bacteria in the anterior intestine (intestinal bulb) were selected to continue the experiment. These larvae were incubated at 28 °C for 24 h. To prepare the larvae for confocal microscopy with a Nikon Eclipse Ti Fluorescence Microscope, they were incubated on ice for 3 min and then fixed in cold 4% PFA for 1 h. Microscopic observation was used to determine the rates of bacterial retention in the gut, and the retention rates were reported as the number bacteria-positive guts divided by the total number of observations. For the image analysis, ImageJ Fiji software was used to construct Z-stacks of the fluorescent images merged with the corresponding bright field images. The results were obtained from 5 replicates (each one using 10-21 larvae per treatment). Statistical analysis (ANOVA) showed significant differences between the groups. Experiments were carried out in accordance with Spanish Institutional Animal Use and Care Committee regulations and with the approval through the Regional Andalusian Government (code A/ES/14/43).

### Ethical approval and informed consent

Experiments were carried out in accordance with Spanish Institutional Animal Use and Care Committee regulations and with the approval through the Regional Andalusian Government (code A/ES/14/43).

The methods were carried out in accordance with the relevant guidelines and regulations.

## Supplementary information

Supplementary info.

## Data Availability

All data generated or analyzed during this study are included in this published article (and its Supplementary Information files).
