# Supplementary material for: Two genomic regions encoding exopolysaccharide production systems have complementary functions in B. cereus multicellularity and host interaction
Source: Sci Rep. 2020 Jan 22;10:1000. doi: 10.1038/s41598-020-57970-3 (PMC6976573; doi:10.1038/s41598-020-57970-3)
Supplement: Supplementary file 1 — Supplementary info. [file 41598_2020_57970_MOESM1_ESM.pdf]

Two genomic regions encoding exopolysaccharide production systems have complementary functions in *B. cereus* multicellularity and host interaction

Caro-Astorga Joaquin, Álvarez-Mena Ana, Hierrezuelo Jesús, Guadix Juan Antonio, Heredia-Ponce Zahira, Arboleda-Estudillo Yohanna, González-Munoz Elena, de Vicente Antonio, and Romero Diego

Supplemental material

Supplemental figures

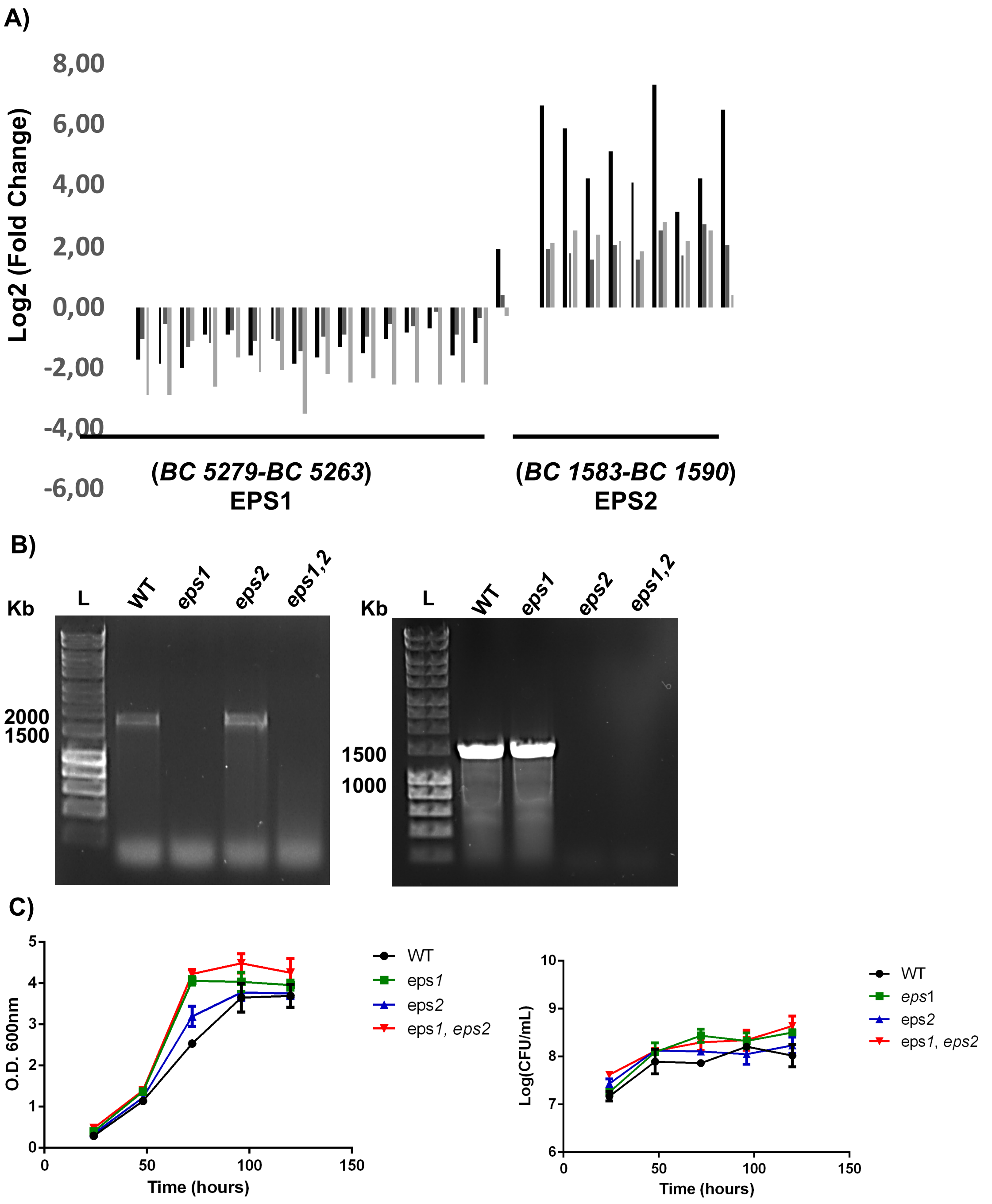

Figure S1. Two genomic regions of *B. cereus* hypothetically committed to production of exopolysaccharides are differentially expressed in biofilm cells. A) Transcriptomic analysis showed the specific overexpression of genes of the *eps2* region but not the *eps1* in biofilm cells compared to floating cells at 24 h (black), 48 h (dark grey) and 72 h (clear grey). B) Validation of the *eps* mutants in *B. cereus* strains. Amplifications correspond to *eps1* (left panel) (1683 bp) and eps2 (right panel) (1419 bp) genomic regions. Sizes (Kb) are indicated. (L) Molecular Ladder. C) Dynamic of growth of *B. cereus* WT and *eps* mutants in the biofilm experimental conditions. Left: Measurement of optical density at 600 nm at 24h, 48h, 72h, 96h and 120h; right: Representation of the Log(CFU/ml) of *Bacillus* cells at these time points.

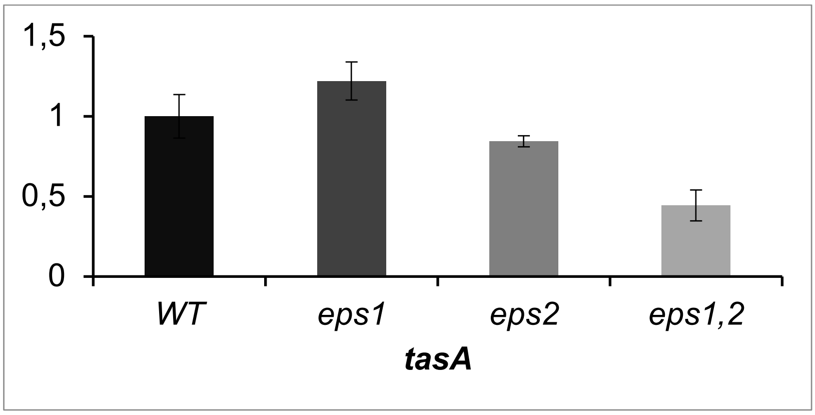

Figure S2. Level of expression of *tasA* (*BC_1279*) in *eps* mutants. Levels of transcripts of tasA were analyzed by Q-RT-PCR. The data were normalized to the level of housekeeping gene *rpoA* and the fold change ratio was calculated from *eps1* (A), *eps2* (B) and *eps1,2* (C) mutants compared to wild-type (dashed line). Statistical analysis, ANOVA with post-hoc paired Student’s test, didn´t show any significant differences between the samples.

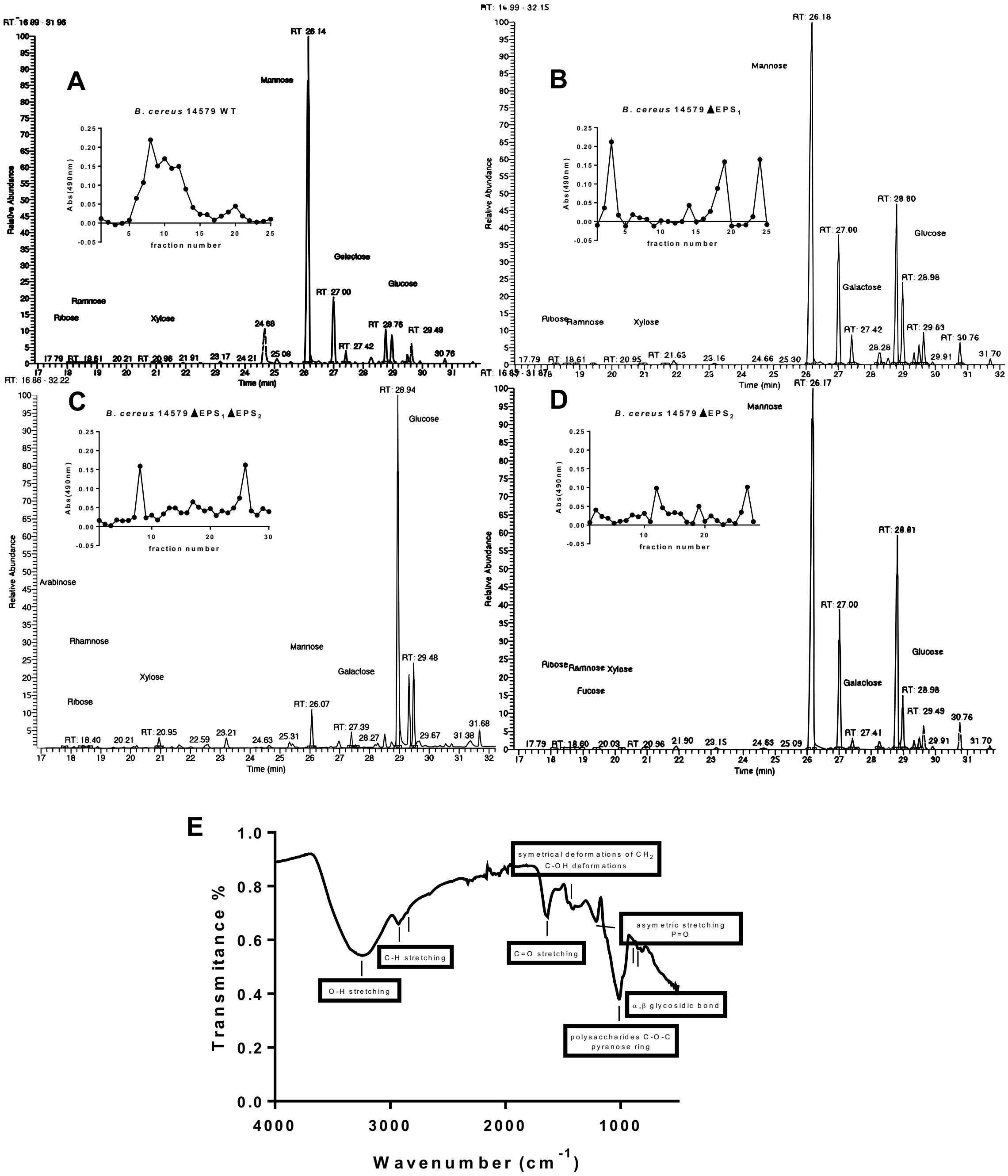

Figure S3. (A-D) Gas chromatograms of the fractions of exopolysaccharide (EPS) at higher molecular weight in gel filtration chromatography of *B. cereus* 14579 and mutants. The absorption at 490 nm of aliquots from the size exclution column is represented above each chromatogram. (E) FT-IR spectrum of the extracted EPS in WT sample and most representative bands.

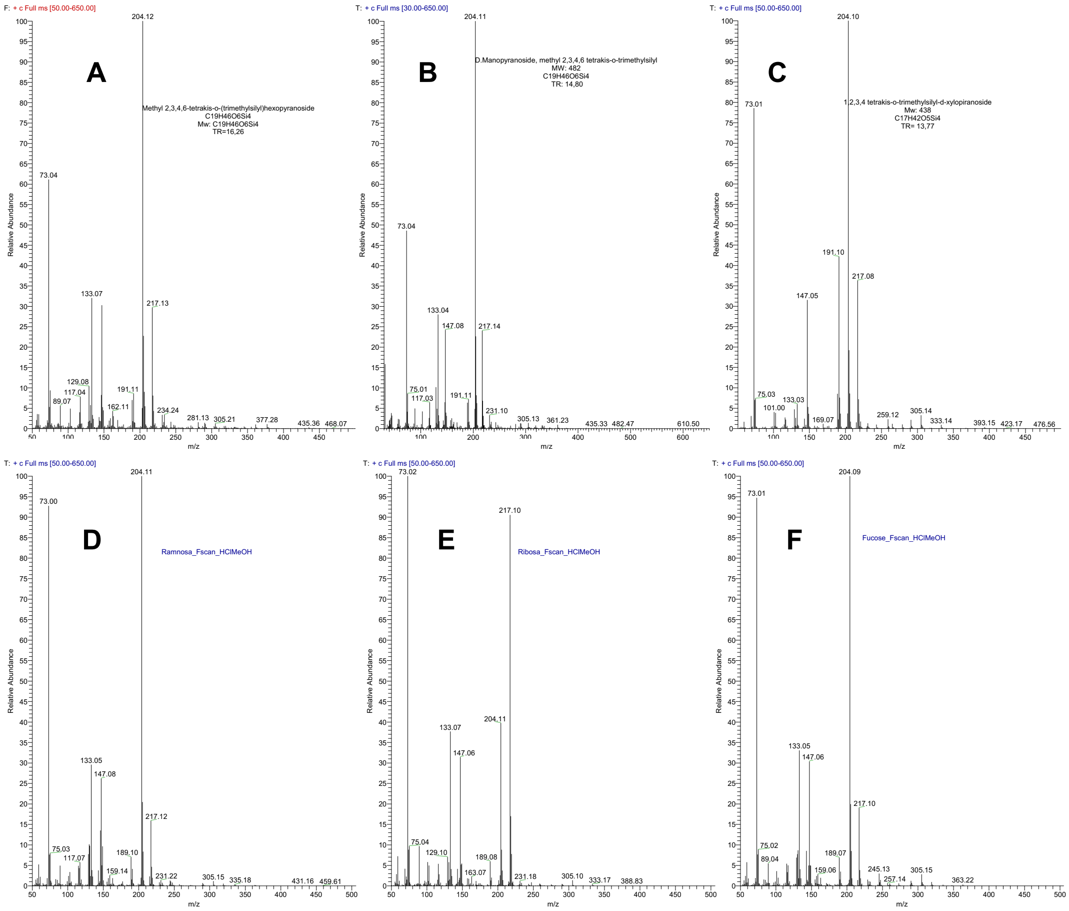

G

Strain		Glucose	Mannose	Galactose	Arabinose	Xilose	Rhamnose	Ribose	Fucose
B. cereus	ATCC14579	+	+	+	-	+	+	+	-
B. cereus	ATCC14579 Δeps1	+	+	+	-	+	+	+	-
B. cereus	ATCC14579 Δeps2	+	+	+	-	+	+	+	+
B. cereus	ATCC14579 Δeps1 Δeps2	+	+	+	-	+	+	+	-

Figure S4. (A-F) Mass spectra of the glucose, mannose, xylose, rhamnose, ribose and fucose derivatives obtained after methanolysis and ionization by electronic impact (EI) in mass spectrometry. (G) Sugar unities identified in the EPS fractions produced by wyld type and mutants strains.

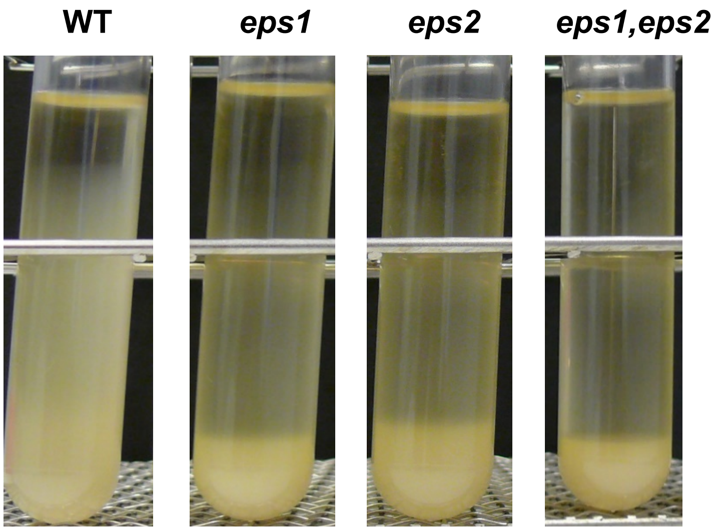

Figure S5. Eps1 and Eps2 prevent cellular auto-aggregation. Pictures of the tubes at 24 h of incubation at 30 ºC and no agitation shows major aggregation of mutants than the WT.

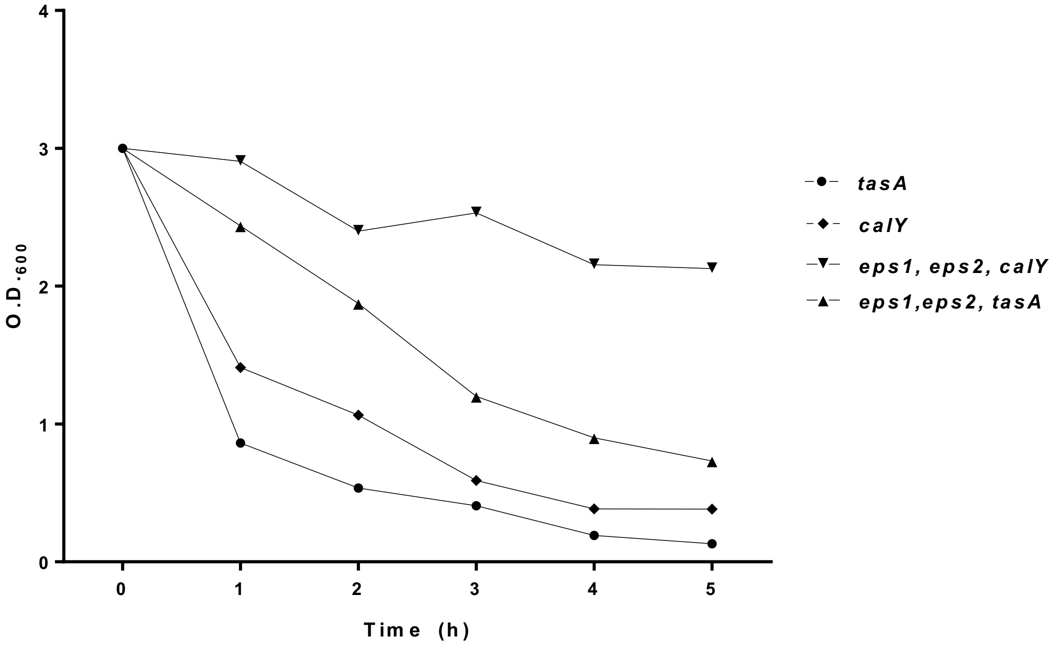

Figure S6. EPS1 and EPS2 interact and affect cell aggregation. OD measures of the air-liquid interface of cell suspensions in static conditions at room temperature. Cultures in TY were incubated at 150 rpm and 30 ºC for 16 h and OD was adjusted to OD=3 before the experiment initiation.

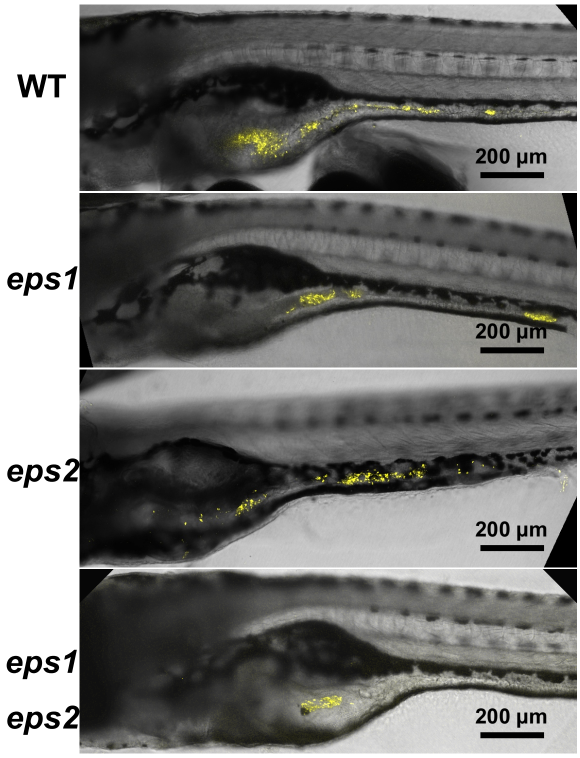

Figure S7. The EPS2 is more relevant for adhesion of *B. cereus* to human epithelial cells and Zebrafish gut. Bacteria localization in zebrafish larvae gut after feeding.

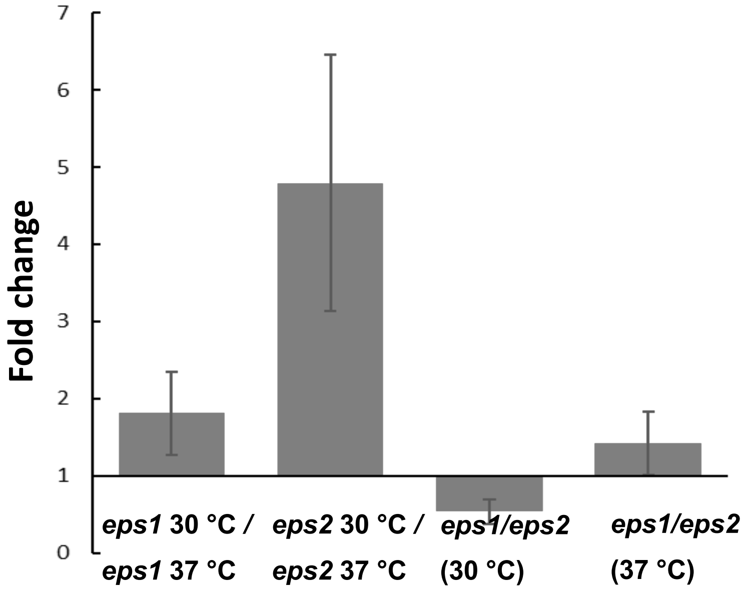

Figure S8. *eps1* and *eps2* expression patterns varie depending on temperature. qRT-PCR analysis was done to determine the relative levels of transcripts of representative locus of the *eps1* or *eps2* genomic regions at 30 ºC and 37 ºC.

Supplemental Tables

Table S1. Bacterial strains used in this study.

Strain	Genotype	Plasmid	Reference
B. cereus ATCC14579			This study
B. cereus ATCC14579	*Δeps1*		This study
B. cereus ATCC14579	*Δeps2*		This study
B. cereus ATCC14579	*Δeps1 Δeps2*		This study
B. cereus ATCC14579		pHCMC02-*Pupp-yfp*	This study
B. cereus ATCC14579	*Δeps1*	pHCMC02-*Pupp-yfp*	This study
B. cereus ATCC14579	*Δeps2*	pHCMC02-*Pupp-yfp*	This study
B. cereus ATCC14579	*Δeps1 Δeps2*	pHCMC02-*Pupp-yfp*	This study
B. cereus ATCC14579	*Δflag*		This study
B. cereus ATCC14579	*Δeps1, Δflag*		This study
B. cereus ATCC14579	*Δeps2, Δflag*		This study
B. cereus ATCC14579	*Δeps1, Δeps2, Δflag*		This study

Table S2. Primes used to mutate the *eps1 (BC5279-5274)* and *eps2(1583-1591* regions and flagellin.

Mutations	Name	Primer sequence
ΔBC_1583–1591	Forward.up.BamHI	AAAAGGATCCGGGATGTTGCATAAGTCGAAC
ΔBC_1583–1591	Reverse.up	ATTGGCATTAATCCAGCAAGGCCACGAGC GATTAAACCATTC
ΔBC_1583–1591	Forward.dw	CCTTGCTGGATTAATGCCAAT
ΔBC_1583–1591	Reverse.dw.NcoI	AAAACCATGGCTCTTT TTCATTACCTATAT CCCACTA
ΔBC_5279-5274	Forward.up.BamHI	AAAAGGATCCTTTGAAAGAACTAAGGCTGA CG
ΔBC_5279-5274	Reverse.up	TGGTTTTCTTTTCTTTCTCCCGAACATAT T
ΔBC_5279-5274	Forward.dw	AAACCATTACGACAATTAATTACACATCAGATGA GTCAAGA GAGTATTAGGAAAACC
ΔBC_5279-5274	Reverse.dw.NcoI	AAAACCATGGATGCAAAGGTAACGTGATT TCATT
Δflag	Flag.up.fw	CGATGCATGCCATGGTACCCTTATTTTGA AACGTCGATATTTAATATTTTTTTAATATATC
	Flag.up.rv	AACACAGTAAGTGCTTGCATATAAGAAAG
	Flag.dw.fw	ATGCAAGCACTTACTGTGTTACGGTCATAAC
	Flag.dw.rv	CTTCTAGAATTCGAGCTCCCGTGGCACAGATTA TGTATC
**Primers to validate mutations**		
eps1	Eps1 Fw	TGTAACGGATGCGCAAATTA
	Eps1 Rv	TTCGCAAGAATTTGTGCATC
eps2	Eps2 Fw	TGTTTTGAGCGGATTTGTTTTGT
	Eps2 Rv	GATTGCTCTGCCAATGTCTTT

Table S3. Primes used for the construction of the plasmid pHCMC02-*Pupp-yfp.*

Name	Primer sequence
Prom.upp.Fw	AAAAAGCTAGCGGATGAAATTGCGTCA
Prom.upp-yfp.Rv	CATAGTAGTTCCTCCTTATGTAGATGATA TTCATGCGTTTGC
yfp.Fw	ACATAAGGAGGAACTACTATGAGT
yfp.Rv	GCGCTCACCCGGGTTATTTGTATAG

Table S4. Primes used for RT-PCR assays.

Name	Primer sequence
BC_1583_fw	TGTTTTGAGCGGATTTGTTTTGT
BC_1583_rv	GTCCACGAGCGATTAAACCA
Interg_83-84_fw	ATTCAGAAAAGGGCGGTGAAAT
Interg_83-84_rv	AGCGGTTAAACAACACATCGT
BC_1584_fw	AAAATCTCGGTTGTGAATACGG
BC_1584_rv	GATTGCTCTGCCAATGTCTTT
Interg_84-85_fw	TCGAGGTGAACATGTCGATG
Interg_84-85_rv	CACCTCTGTCCTTCGTTGAA
BC_1585_fw	ACATACAGGGACGCCTTTAGT
BC_1585_rv	AACCACTCCATCGCATTTCC
Interg_85-86_fw	TTGAATGTGGGGGTAACGAT
Interg_85-86_rv	CAAACAAGCGTTTTACACTTCG
BC_1586_fw	CCCTAGACCAGAGAGAGAGTTTT
BC_1586_rv	CCCTCCATTTATTTGTGCCCA
Interg_86-87_fw	TGAACGGTAATGGTGCAAGA
Interg_86-87_rv	AAACTGACGGCCTGAAGAAG
BC_1587_fw	GAATGCAGTAGAACCACTCCAAA
BC_1587_rv	CGTCGTTAAACCGTCAGCAA
Interg_87-88_fw	CGCAAAAAGGATTATATTACGAA
Interg_87-88_rv	GAAGAATAATCCATCCCATTGA
BC_1588_fw	CGCAGCATACTTTGTTTCGTG
BC_1588_rv	ACCATCTATCTTTCCCCTTCCTT
Interg_88-89_fw	CGCAGCATACTTTGTTTCGTG
Interg_88-89_rv	GAAGAATAATCCATCCATCCATCCCATTGA
BC_1589_fw	TGTTCAACCGACCAATCTGC
BC_1589_rv	TGCGGCAACCATATATCATCAC
Interg_89-90_fw	GAAGAATAATCCATCCATCCATCCCATTGA
Interg_89-90_rv	GCCATCGCTTCAATTACTACCA
BC_1590_fw	ATTTCAGAGCCGCATGAGTTT
BC_1590_rv	GCCATCGCTTCAATTACTACCA
Interg_90-91_fw	TTTAATATCTGAATATTGCGAAT
Interg_90-91_rv	AATTTGCAGGGTTACGGTAGG
BC_1591_fw	TTTAATATCTGAATATTGCGAAT
BC_1591_rv	TTGGACCTGCTGCCATTAAG
BC_5279_fw	TCGGACAACAAGGAAAGAAAGT
BC_5279_rv	TTGGACCTGCTGCCATTAAG
Interg_79-78_fw	CTCGGAGTCGTTTTGAATGAT
Interg_79-78_rv	CGAGGATCATTGCTAAACGTT
BC_5278_fw	GCTTCAGTTGGTCTTGCATTCT
BC_5278_rv	TCTTGATGATGGAGCGTGTGA
Interg_78-77_fw	GCTTCAGTTGGTCTTGCATTCT
Interg_78-77_rv	AGAAGCATCACATACATTTGCCA
BC_5277_fw	ATCCCACCAAATCCAGCAGA
BC_5277_rv	AGAAGCATCACATACATTTGCCA

Interg_77-76_fw	TATTACTACGGTGCAAACTAG
Interg_77-76_rv	GTGACAATGTAAATCTATCAT
BC_5276_fw	GCACAAAAAGCCGCTTCAGA
BC_5276_rv	TAACCTGACTTCTTGCCCCG
Interg_76-75_fw	TGGTGATGTCTCCTAACCGT
Interg_76-75_rv	TAATGAGAGCCGTCTTCGATA
BC_5275_fw	GCAAGGAACAGCACCATCAA
BC_5275_rv	CCCGCACCTGTTTGTTGATT
Interg_75-74_fw	TGTTGGGGAGAAGTTTGGAT
Interg_75-74_rv	ACTCAATATACCTGTTCCCTTCT
BC_5274_fw	CTAGGGAGAGAGCCTGTTCA
BC_5274_rv	CCCCAGCACCCGTTATTAAA
Interg_74-73_fw	CCTTATTAGATTTTGCGAATA
Interg_74-73_rv	GTCTGGTGGAGAAAACGGGA
BC_5273_fw	CCGTTTTCTCCACCAGACATT
BC_5273_rv	ACCGCTTTATTTGTCCCAACA
Interg_73-72_fw	AATGAAATCACGTTACCTTTGCA
Interg_73-72_rv	TGAACTGCTGCAACTCCTAG
BC_5272_fw	ACAAGGGCAAAGAGGGGTAA
BC_5272_rv	CCAAGTTTCTCTATCGGACGC
Interg_72-71_fw	CGTCCAGTAAATGGTAAAAATGA
Interg_72-71_rv	TAGAAAACCCGTTCCACCAG
BC_5271_fw	TTGGCCTGTGAGCATATTGG
BC_5271_rv	TCACCATGAAATGCTTGTCTGA
Interg_71-70_fw	TTGGCCTGTGAGCATATTGG
Interg_71-70_rv	CAGAGGCTGCCGTTAAGAAT
BC_5270_fw	ACAATGGAGCAACGTGTGAA
BC_5270_rv	CAGAGGCTGCCGTTAAGAAT
Interg_70-69_fw	ACAATGGAGCAACGTGTGAA
Interg_70-69_rv	ACATCCATTGCACGACTTTCA
BC_5269_fw	GGACTGTCCCTGAAAATGTTGT
BC_5269_rv	ACATCCATTGCACGACTTTCA
Interg_69-68_fw	CTTGGGAAGTCTAGTTCTGGG
Interg_69-68_rv	TGCAAGTCCTATTACCCTCCT
BC_5268_fw	TTAGGAGCCTGGATGTGTCT
BC_5268_rv	CGTTGTCGTTTGAACCCTTTT
Interg_68-67_fw	AAAAAAGGGTTCAAACGACAAC
Interg_68-67_rv	TCTGTAGATCCGTCATTTACCA
BC_5267_fw	TTGGTAAATGACGGATCTACAGA
BC_5267_rv	TCAATCCTACATTTCTCGCAGA
Interg_67-66_fw	GAATGCTAAACCAAGTAACTAA
Interg_67-66_rv	TAGCAATAAATGTATAAGAG
BC_5266_fw	TTTCCTGCTTTACGTCGTACATC
BC_5266_rv	CGGTAACCACGCTGATAGGA
Interg_66-65_fw	ATGCTCATGAAATCTAAATGA
Interg_66-65_rv	AATAGGATTTTCTTTTTCAT

BC_5265_fw	CAGTTGGTGGGGTAGATGTCA
BC_5265_rv	AACAGCTTGCATCACTTGGC
Interg_65-64_fw	GAGCGCATCTTGAAGTGACC
Interg_65-64_rv	CGCTCACAATCGAAGCAACA
BC_5264_fw	GCAACAGGACAAACGAGTGA
BC_5264_rv	TGCCTTTTTATCCTCAGCAGC
Interg_64-63_fw	GGACATGATGTTTGGGCGAA
Interg_64-63_rv	TATAGCCAGCTCCACCACAG
BC_5263_fw	CTGTGGTGGAGCTGGCTATA
BC_5263_rv	CGCCTTCCGTAATTGCATCT
